# P-822. Unraveling the Landscape of Staphylococcus aureus Bloodstream Infection: Insights from a Single-Center Study

**DOI:** 10.1093/ofid/ofae631.1014

**Published:** 2025-01-29

**Authors:** Santhanam Naguthevar, Navneet Kaur, T R Neetha, Yash Khatod, Tejasvi kanagiri, Bhuvanesh Kumar, Akshatha Ravindra, Vibhor Tak, Deepak Kumar, Durga Shankar Meena, Naresh Kumar Midha

**Affiliations:** AIIMS JODHPUR, Jodhpur, Rajasthan, India; All India Institute of Medical Sciences, Jodhpur, Jodhpur, Rajasthan, India; AIIMS Jodhpur, Jodhpur, Rajasthan, India; AIIMS Jodhpur, Jodhpur, Rajasthan, India; AIIMS, Jodhpur, Jodhpur, Rajasthan, India; AIIMS, Jodhpur, Jodhpur, Rajasthan, India; AIIMS Jodhpur, Jodhpur, Rajasthan, India; AIIMS Jodhpur, Jodhpur, Rajasthan, India; All India Institute of Medical Sciences, Jodhpur (India), Jodhpur, Rajasthan, India; AIIMS, Jodhpur, Rajasthan, India; AIIMS, Jodhpur, Rajasthan, India

## Abstract

**Background:**

*Staphylococcus aureus* significantly impacts human health, with global prevalence ranging from 13% to 74% and 37% in India. By delving into the intricacies of this bloodstream infection, we aim to provide valuable insights into its manifestations, risk factors, and antibiotic sensitivities, crucial for guiding tailored treatment strategies and optimizing patient outcomes.

**Methods:**

Between February 2022 and January 2024, a prospective observational study was conducted at AIIMS, Jodhpur, India, encompassing all blood culture-positive Staphylococcus aureus patients, with comprehensive collection of clinical, epidemiological, microbiological, and outcome data.

**Results:**

In our study, we observed a diverse demographic distribution among Staphylococcus aureus bloodstream infection cases. The most common age group affected was 1-20 years, comprising 29.82% of cases, followed closely by individuals aged 21-40 years (24%), 41-60 years (23.27%), and those over 60 years old (22.91%).

Clinical manifestations varied, with Hospital-Acquired Pneumonia (HAP) being the most common syndrome at 31.27%, followed closely by Central Line-Associated Bloodstream Infection (CLABSI) at 27.27%. Other prevalent syndromes included Community-Acquired Pneumonia (CAP; 17.45%), Bone & Joint infections (22.54%), Meningitis (10.18%), Urinary Tract Infection (UTI; 2.54%), Skin and Soft Tissue Infections (SSTI; 4%), and Endocarditis (4%).

Regarding Staphylococcus aureus strain types, Methicillin-Sensitive Staphylococcus aureus (MSSA) constituted the majority of cases at 55.64%, followed by Methicillin-Resistant Staphylococcus aureus (MRSA) at 42.54%, and Vancomycin-Resistant Staphylococcus aureus (VRSA) at 1.82%.

Among the recorded outcomes, the majority of patients (78.54%) showed improvement following treatment, while 16.36% of patients succumbed to the infection. Additionally, 5.09% of patients were lost to follow-up.

**Conclusion:**

Despite progress, many patients succumbed or were lost, highlighting the need for vigilant monitoring and enhanced treatments. Our study underscores the urgency to improve outcomes and curb Staphylococcus aureus infections.

**Disclosures:**

**All Authors**: No reported disclosures

